# Void characteristics and tortuosity of calcium silicate-based cements for regenerative endodontics: a micro-computed tomography analysis

**DOI:** 10.1186/s12903-021-01940-2

**Published:** 2021-11-08

**Authors:** Sang-Yeop Chung, Yun Hyeong Kim, Yong Kwon Chae, Su-Sung Jo, Sung Chul Choi, Ok Hyung Nam

**Affiliations:** 1 Department of Civil and Environmental Engineering, Sejong University, Seoul, Republic of Korea; 2 Department of Dentistry, Graduate School, Kyung Hee University, Seoul, Republic of Korea; 3 Department of Pediatric Dentistry, School of Dentistry, Kyung Hee University, Kyungheedae-Ro 26, Dongdaemoon-Gu, Seoul, 02447 Republic of Korea

**Keywords:** Calcium silicate cement, Dental materials, Regenerative endodontic protocol, Sealing ability, Tortuosity

## Abstract

**Background:**

Internal voids of materials can serve a hub for microorganism and affect the sealing ability. This study aimed to evaluate the sealing performance of calcium silicate-based cements in immature teeth treated with regenerative endodontics.

**Methods:**

Twenty single root canals from immature permanent premolars were prepared using regenerative endodontic protocols. The root canals were randomly divided into two groups and sealed with mineral trioxide aggregate (MTA) and Biodentine (BD). The teeth were kept in humid environment for 7 days and scanned using micro-computed tomography. The voids within the cements were segmented and visualized using image processing, incorporating the modified Otsu algorithm. The porosity of each sample was also calculated as the ratio between the number of voxels of voids and the volume of the cements. Tortuosity was also calculated using the A-star algorithm.

**Results:**

Voids larger than 70 μm were predominantly observed in the top and interfacial surface of cements. The others were evenly distributed. MTA and BD showed the same level of porosity and tortuosity at interfacial surfaces. In inner surfaces, MTA showed more less porosity and tortuosity compared to BD (*p* < 0.05).

**Conclusions:**

There were no differences in sealing performance between MTA and BD.

## Background

Anatomy of immature permanent teeth is characterized by wide root canals with open apex and thin dentinal walls. Thus, endodontic treatment of necrotic immature permanent teeth is challenging because of the unique anatomy [1]. Recently, regenerative endodontic procedures (REPs) have been suggested as an alternative approach for the treatment of necrotic immature permanent teeth. The clinical protocol of REPs includes disinfection, provocation of bleeding, and a coronal seal [2]. Ideal situation for REPs requires a blood clot below the cementoenamel junction (CEJ) and sealing with a biocompatible material [3]. The material used should provide a tight seal against microorganism as well as induce mineral formation [3, 4]. Calcium silicate cements, such as mineral trioxide aggregate (MTA) and Biodentine (BD), are recognized as suitable sealing materials for this purpose [5].

Clinically, a void-free seal is difficult to be accomplished. Residual microorganisms can re-grow within the voids. The voids within the materials are less critical in this topic, as they are trapped in an isolated environment. However, voids at the interface between the material and the dentinal wall may serve as hubs for microorganism, leading to microleakage [6]. Coronal microleakage has been regarded as potential cause of treatment failure after endodontic treatment. A previous in vivo study demonstrated that coronal microleakage could lead to periapical inflammation [7]. And these voids could be a cause of re-infection as they are in contact with potentially infected canal walls [8]. Furthermore, such voids act as structural defects that can serve as potential stress concentration points, resulting in the reduction of strength [6, 9]. Therefore, the sealing ability of the materials can be influenced by void formation.

To evaluate sealing ability, leakage studies using dye or bacteria, scanning electron microscopy (SEM), and animal models have been employed [10, 11]. Recently, for a more detailed analysis of the sealing ability, micro-computed tomography (micro-CT) has been used to effectively reveal internal void structure without damaging the samples. Studies using micro-CT have successfully demonstrated the characteristics of materials, particularly the pore structures, and their effects on the sealing ability [12–14]. Several studies have evaluated the sealing ability of calcium silicate cements in various clinical situations. However, few studies have been conducted on sealing materials used in REPs. Therefore, this study aimed to evaluate void structures of MTA and BD, which are dominant factors in determining the sealing of the cement-based materials. In addition, tortuosity, a parameter that can represent a percolation characteristic, was examined, and its correlation with the sealing ability was also discussed. The null hypothesis of this study was that sealing performance of MTA was superior to BD.

## Methods

### Sample preparation

This study proposal was reviewed and approved by the Ethics Committee of Kyung Hee University Dental Hospital, Kyung Hee University (KH-D20-038). This study followed the CRIS guidelines for in-vitro studies, as discussed in the 2014 concept note [15]. Sample size calculation was performed using G* Power 3.1.9.4 program (Heinrich Heine, University Düsseldorf, Düsseldorf, Germany). The size was estimated with α of 0.05 and β power of 0.95. The effective size was calculated based on a previous study [16]. The required sample size was 10 samples per group.

Twenty immature premolars with a single root canal were collected. Teeth with any damage to root surfaces, such as root fractures or cracks, were excluded. The teeth were immersed in 4% sodium hypochlorite (NaOCl) for 24 h and subsequently in 0.1% thymol at 4 °C. The teeth were randomly divided into two groups (*n* = 10); one group was sealed with ProRoot MTA (Dentsply, Tulsa Dental, JC, USA) and the other with BD (Septodont, Saint-Maur-des-fossés, France). The clinical procedure of REPs was performed in accordance to the guidelines of revitalization procedures by the European Society of Endodontology, except pulp extirpation and inducing blood clot because pulps are already removed by 4% NaOCl and 0.1% Thymol during storage [17]. An endodontic access opening was prepared. The canals were irrigated with 2% NaOCl (20 mL, 5 min) and sterile physiological saline (5 mL), following which they were dried using paper points. Thereafter CollaCote (Integra LifeSciences Corp, Plainsboro, NJ, USA) was placed approximately 4 mm below the CEJ. The materials were applied 3–4 mm thickness over the CollaCote with gentle vertical pressure via hand plugger (S-Kondenser; Obtura Corporation, Spartan, USA). The materials were mixed according to the manufacturer’s instructions. The cavity was closed using a Caviton (GC, Tokyo, Japan). Finally, the teeth were maintained in Hank’s balanced salt solution in sealed containers at 37 °C for seven days as previously described [6, 12]. All experiment procedures were performed by one pediatric dental specialist.

### Micro-CT imaging process

This study employed micro-CT (Skyscan 1172, Bruker micro-CT, Kontich, Belgium) to analyze the three-dimensional (3D) microstructure of the cements. The scanning parameters were 100 kV, 100 μA, and 1180 ms. With the micro-CT device, the tooth specimen was scanned to obtain sequential 2D images of the internal cross-sections, which were then merged and converted into 3D images. The initially reconstructed images for the study were composed of pixels with a size of 5.9 μm.

**Fig. 1:**
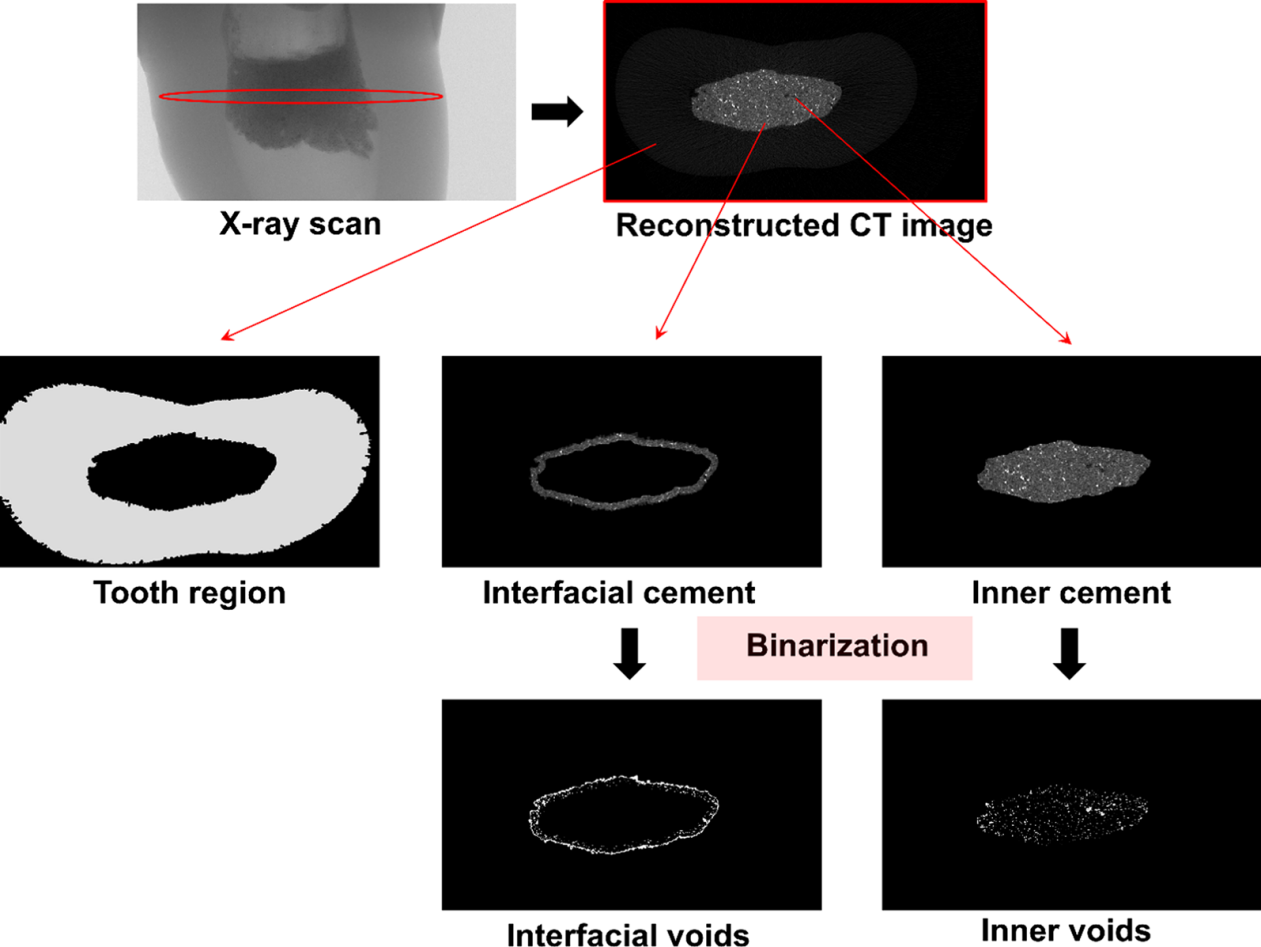
Segmentation and binarization of micro-CT image for a tooth sample. The reconstructed micro-CT image, produced with an X-ray scan, was an 8-bit grayscale image with a resolution of 1600 × 900, and the pixel size of the micro-CT images was 5.9 μm. In case of 8-bit grayscale images, their values range from 0 to 255 (total of 256 values), and the pixel value was determined based on the relative density of components. The region in black represents voids, while the area in bright color can be considered as the cement material inside the tooth

Briefly, a 3D image of the specimen was obtained by subsequent stacking of the binary images, and the produced image was visualized and binarized to analyze the characteristics of the voids (Fig. 1). To characterize the spatial distribution of voids, image processing was conducted to separately analyze the void characteristics at the interface between the canal wall and the cement, as well as the inner cement. First, the cement part was segmented from the teeth using a multi-Otsu threshold algorithm [18], and the cement part was classified into two parts: interfacial and inner cement. For the classification, the boundary of the cement was outlined by recognizing the internal geometry of the targeted tooth, and a region of 40 pixels (approximately 236 μm) from the canal wall was set as the interfacial cement; the cement part excluding the interfacial region was defined as the inner cement. The same procedure was applied to all the samples.

**Fig. 2:**
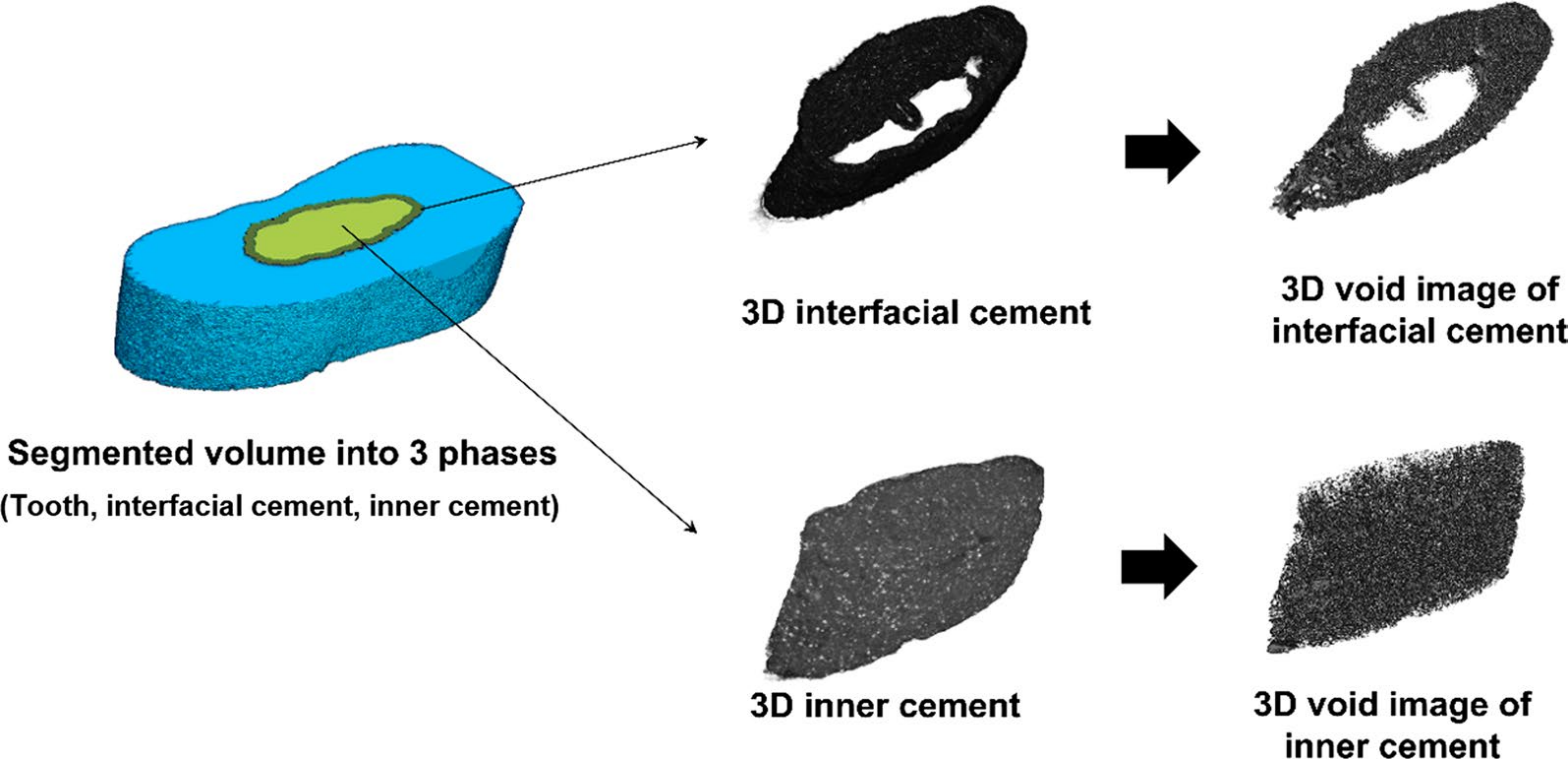
Segmentation of pores for interfacial and inner cement regions in 3D volume

For a more effective analysis, the void characteristics in both the interfacial and inner cements were investigated using binarized micro-CT images. Binarization of each sample was conducted based on the histogram of grayscale micro-CT images. For this purpose, a proper threshold value needed to be selected, and pixels with values lower than the threshold were considered as voids (Fig. 2). To select the threshold, the modified Otsu method and a modified watershed algorithm [19] were adopted, and the image processing was carried out using an imaging toolbox in MATLAB R2020b (Mathworks, Inc., Natick, MA, USA).

### Void structure analysis

The void size distributions of the specimens were investigated to determine the spatial distribution of the voids according to their sizes. Next, the porosity of the interface and the inner parts was computed as the number of void voxels compared to the volume of the interface/inner cement area. For this study, only voids larger than 5.9 μm were considered for the calculation by considering the resolution of the image used.

### Tortuosity analysis

To investigate the characteristics of the spatial distribution of voids, tortuosity, a quantitative index of void characteristics, was estimated in the interfacial and inner cement of MTA and BD. As an index to express the degree of curvature of a certain space, tortuosity can be employed to describe and analyze the connectivity of voids within materials [20]. In particular, tortuosity can be used as a parameter to describe a percolation characteristic, which has a strong relationship with the sealing and durability of the materials [21]. Tortuosity is defined as the ratio of the path length, as in the following formula:

$$\tau =\frac{{L}_{A}}{{L}_{s}}$$

Here, τ refers to the tortuosity, ${L}_{A}$ denotes the actual distance of the void path, and ${L}_{s}$ is the shortest distance between the starting and end points on the void path.

**Fig. 3:**
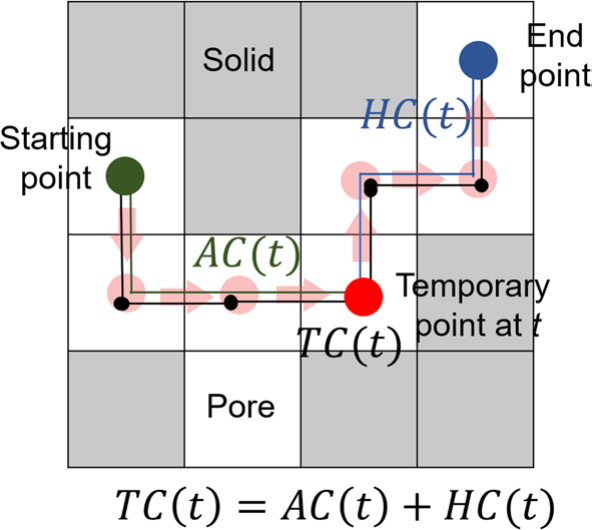
Schematic of the A-start algorithm. AC(t) denotes the actual distance between the starting point and a temporary point, while HC(t) is the distance between the temporary and end points at iteration process t. The total cost TC(t) is computed by adding the two functions

In this study, the A-star algorithm was used to calculate ${L}_{A}$, the actual distance of the pore path (Fig. 3). This algorithm is used to find the shortest void path from an arbitrary starting point to the end point [22]. The shortest path was calculated using two functions: AC(t) and HC(t). First, AC(t), the function to express the actual distance from the selected starting point to a temporary point (t) within voids, was calculated. The heuristic estimate (HC(t)) from the temporary to the end points was then subsequently added with AC(t), and the total sum of the functions, TC(t), was computed. For the calculation of AC(t), the vertical, horizontal, and diagonal distances from the start point to the temporary points were considered, and the temporary point was moved from the start point to the end point by considering the shortest function values at each point. Simultaneously, HC(t) was calculated considering only the horizontal and vertical distances from the temporary point, this approach being known as the Manhattan distance. The sum of the functions, TC(t), was calculated iteratively by moving the temporary point and considering the minimum value of the functions; the path of the temporary point was set as ${L}_{A}$. The tortuosity of the interfacial and inner cement was computed for all specimens to describe the complexity of the void structure within each material and each section.

### Statistical analysis

Data were analyzed using SPSS version 20.0 (SPSS Inc., Chicago, IL, USA). The normality test was performed using the Shapiro–Wilk test, which revealed that the data were normally distributed. Thus, the results were analyzed using the independent T-test. P-values < 0.05 were considered to indicate statistical significance.

## Results

**Fig. 4:**
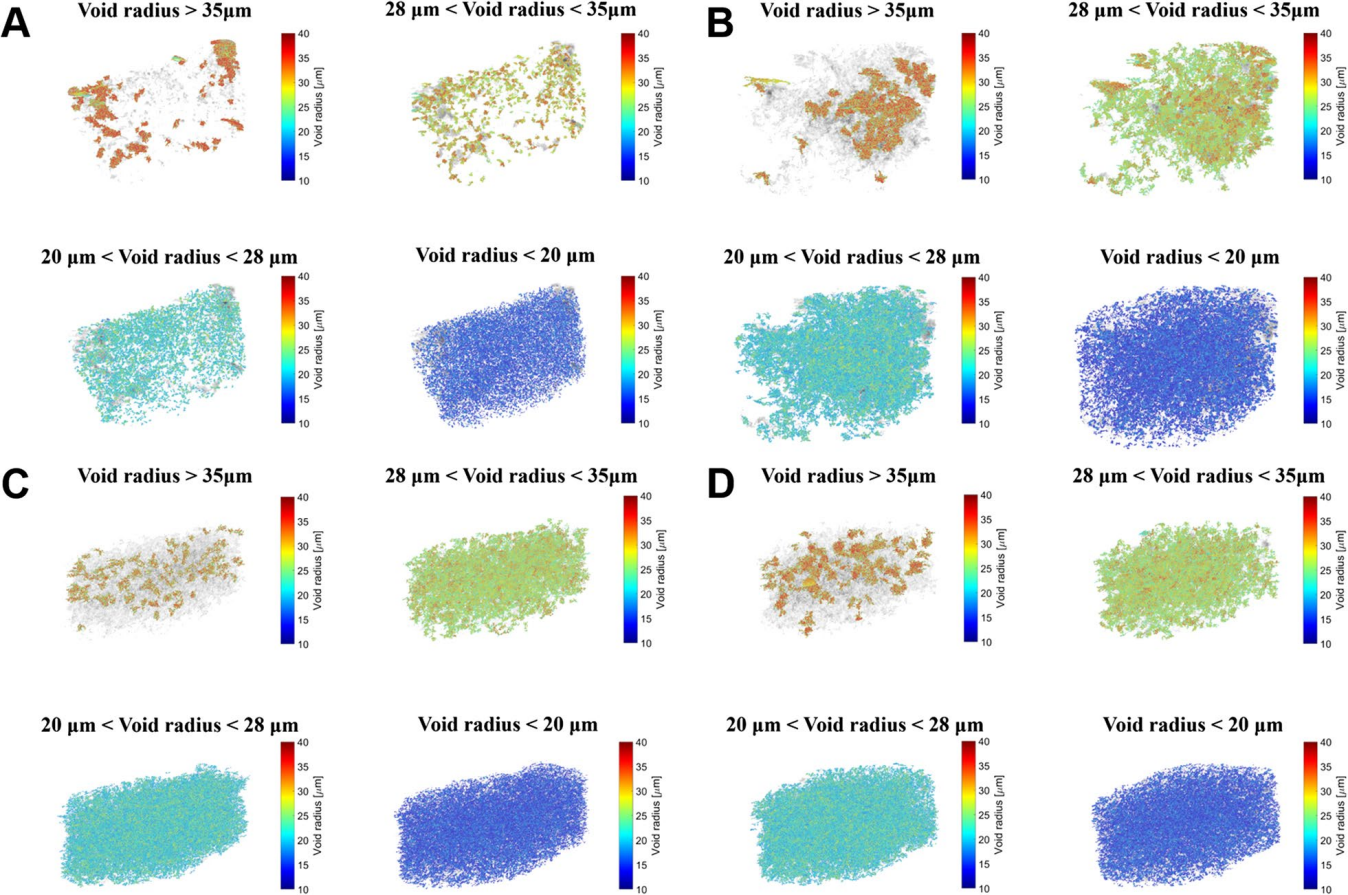
A 3D void distribution of the samples according to their size. Voids are classified into 4 categories: larger than 35 μm, 28–35 μm, 20–28 μm, and smaller than 20 μm. **A**, **B** Representative images of void distribution of MTA samples, **C**, **D** Representative images of void distribution of BD samples. MTA, mineral trioxide aggregate; BD, Biodentine

Figure 4 shows the 3D distribution of the voids in MTA and BD groups. In both the groups, relatively small voids with a radius of < 28 μm were evenly distributed throughout the specimen. However, relatively large voids with a radius > 35 μm were concentrated in the upper part of the specimen or the interface.

The porosities of the interfacial and inner surfaces of each specimen as obtained following a quantitative analysis of voids between MTA and BD are shown in Table 1. There were no significant differences between MTA and BD (*p* > 0.05) with regards to the interfacial surfaces between the cement and canal wall. However, teeth in BD group showed higher porosity in the inner surface compared to those in MTA group (*p* < 0.05).

**Table 1:** Comparison of porosity of mineral trioxide aggregate (MTA) and Biodentine (BD)

	Porosity (%)		*P*-value
	MTA	BD	
Interfacial	22.95 ± 8.04	30.28 ± 7.63	0.05
Inner	14.50 ± 6.60	23.66 ± 4.74	0.02^*^

*P*-values from independent T-test

^*^Statistically significant (*p* < 0.05)

**Fig. 5:**
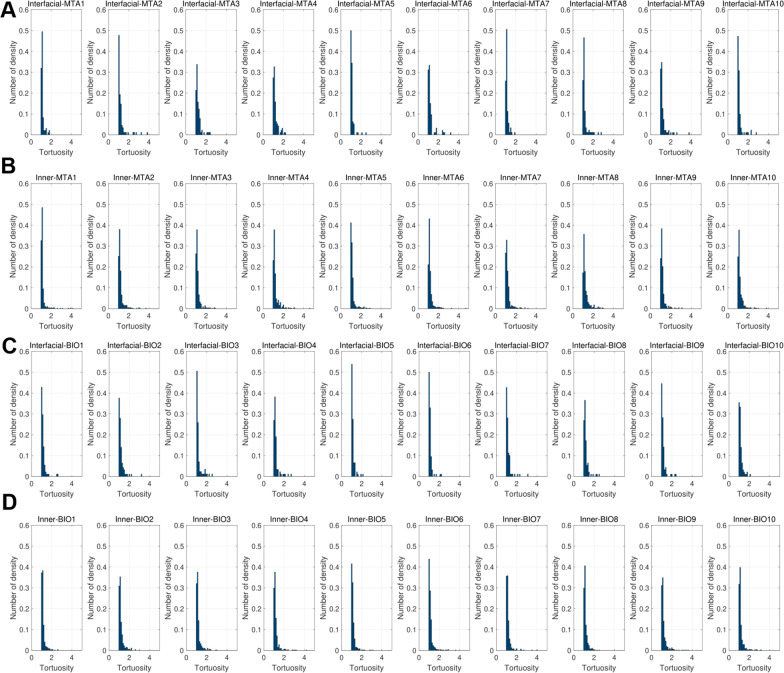
Tortuosity distribution of the samples. **A** Tortuosity of MTA at interfacial surface, **B** tortuosity of MTA at inner surface, **C** tortuosity of BD at interfacial surface, **D** tortuosity of BD at inner surface. MTA, mineral trioxide aggregate; BD, Biodentine

The tortuosity analysis showed similar results (Table 2); with significant differences only being observed at the inner surfaces (*p* < 0.05). The tortuosity distribution is depicted in Fig. 5.

**Table 2:** Comparison of tortuosity of mineral trioxide aggregate (MTA) and Biodentine (BD)

	Tortuosity		*P*-value
	MTA	BD	
Interfacial	1.18 ± 0.05	1.15 ± 0.03	0.16
Inner	1.19 ± 0.03	1.16 ± 0.02	0.03^*^

*P*-values from independent T-test

^*^Statistically significant (*p* < 0.05)

## Discussion

This study evaluated the 3D void structures of MTA and BD using micro-CT analysis, when MTA and BD are used as sealing materials in REPs. A tortuosity analysis was also employed to propose a new approach for assessing the sealing ability of dental cements.

Both MTA and BD showed different 3D void distributions depending on the void size in this study. Relatively large voids, particularly those larger than 30 μm, were clustered mainly in the upper part of the specimen and the interfacial surface with the canal wall, whereas smaller ones were evenly distributed throughout the specimen. These findings are in good agreement with that of a previous study which evaluated voids following hardening with insulating concrete cement paste [23]. The previous study showed that voids with radius larger than 50 μm were clustered mainly in the upper part of the specimen, which was attributed to the bleeding phenomenon during the setting process [24]. The study attributed this finding to the difference in specific gravity between water and voids, which may have resulted in a weaker strength and hardness of these parts, due to large voids affecting the physical properties of the cement-based materials. Milanovic et al. [6] reported that coronal voids may serve as a reservoir for growth of microorganisms. They demonstrated that the effect of coronal voids is extremely limited as voids can be removed during restoration, following adhesive cementation/restoration. A previous study documented that MTA constantly induces the initial precipitation of amorphous calcium phosphate after setting, resulting in the formation of a tag-like structure at the interface between the cement and canal wall after 2 months [25]. Another study showed that both MTA and BD have similar potential to induce biomineralization [26]. Biomineralization largely contributes sealing ability of MTA and BD [27]. However, the effects of tag-like structures were not confirmed in this study, probably because of the study design.

In this study, the porosity of MTA and BD was approximately 14.5–30.3%. These values were higher than those reported in other micro-CT studies that compared porosity after placing MTA and BD into silicone molds [28, 29]. The difference may be attributed to the different experimental conditions, since the application and compaction method employed, and the thickness of cement may affect porosity. BD showed a higher porosity in the inner surface than MTA, which may be due to the different setting times in the early hydration process. As BD allows a shorter setting time, it can have more voids on its inner surface. This finding indicates that MTA possesses a denser structure than BD as previously reported [30]. Conversely, MTA and BD did not show a significant difference in porosity at the interfacial surface. This finding is in line with the results of a previous study that evaluated the 3D morphology of MTA and BD following application of root-end filling materials [16].

To quantify the sealing ability of MTA and BD, tortuosity analysis was conducted. Tortuosity is an indicator of the shortest path in which fluid flows in the specimen, which represents the percolation and sealing ability of materials [20, 31]. The minimum value of the tortuosity is 1, which indicates that the line formed by connecting any point in the void cluster is almost linear without any curvature. In other words, a high tortuosity value would imply that the voids are connected in a complicated pattern in the specimen, denoting difficulties in the diffusion of moisture and fluid, and indicating a relatively low possibility of being impacted by an external environment [32]. Interestingly, there were no significant differences in tortuosity between MTA and BD at the interfacial surface. This reflects that the interfacial surfaces of MTA and BD show similar void connectivity and indicates that both MTA and BD can present similar sealing abilities when used for REPs. A randomized clinical study demonstrated that both MTA and BD were acceptable to create a tight coronal seal [33]. However, there has been controversy about sealing ability between MTA and BD in the previous studies. Cechella et al. [34] reported that BD had a lower level of sealing than MTA when they used for apical plugs. A previous leakage study reported that BD showed superior sealing ability as a root-end filling material compared to MTA by assessing the depth and volume of the penetrated silver nitrate [35]. A recent systematic review regarding sealing ability of root filling materials showed BD has superior sealing ability against MTA [36].

In this study, a 3D analysis of the void structures based on micro-CT images facilitated a more precise and effective evaluation of the spatial characteristics of MTA and BD. However, this study has a few limitations. First, a relatively short-term evaluation was conducted; thus, the effects of biomineralization on the sealing ability were excluded. Second, the normalization of the root canal space was insufficient. Next, MTA and BD have different radiopacifiers that affect the setting and hydration reactions [37, 38]. Thus, these differences may affect void distribution. Finally, the effect of several body fluids such as blood, exudate or saliva were not considered because the experiment was conducted extraorally with extracted human teeth.

## Conclusions

In conclusion, this study evaluated the 3D void structure of MTA and BD used in REPs. This study confirmed that MTA is composed of a denser internal structure than BD and that there are no differences in the sealing abilities of MTA and BD.

## Data Availability

All data generated or analyzed from this study are included in this published article.

## Abbreviations

REPs: Regenerative endodontic procedures
CEJ: Cementoenamel junction
MTA: Mineral trioxide aggregate
BD: Biodentine
SEM: Scanning electron microscopy
micro-CT: Micro-computed tomography
NaOCl: Sodium hypochlorite
3D: Three-dimensional
